# Trends and inequalities in insecticide-treated net use among children under five in Ghana, 2003–2022: analysis of Demographic and Health Surveys using the WHO Health Equity Assessment Toolkit (HEAT)

**DOI:** 10.1186/s41182-025-00869-4

**Published:** 2025-12-05

**Authors:** Amidu Alhassan, Patience Fakornam Doe, Mustapha Amoadu, Augustus Osborne

**Affiliations:** 1 Department of Adult Health, School of Nursing and Midwifery, College of Health and Allied Sciences, University of Cape Coast, Cape Coast, Ghana; 2 Department of Public Health, School of Nursing and Midwifery, College of Health and Allied Sciences, University of Cape Coast, Cape Coast, Ghana; 3 Biomedical and Clinical Research Centre, College of Health and Allied Sciences, University of Cape Coast, Cape Coast, Ghana; 4 Institute for Development, Western Area, Freetown, Sierra Leone; 5 Department of Nursing, School of Biomedical Engineering and Allied Health Sciences, All Nations University, Koforidua, Ghana

**Keywords:** Insecticide-treated nets, Malaria prevention, Inequality, Children under five, Ghana

## Abstract

**Background:**

Malaria is a leading cause of morbidity and mortality among children under five in sub-Saharan Africa, especially in Ghana. Insecticide-treated nets (ITNs) are among the most effective prevention tools, yet disparities in use persist, undermining equitable malaria control. This study examined trends and inequalities in ITN use among Ghanaian children under five from 2003 to 2022.

**Methods:**

A cross-sectional secondary analysis was conducted using six rounds of the Ghana Demographic and Health Surveys (2003–2022). The outcome variable was defined as whether a child had slept under an ITN on the night preceding the survey. Equity stratifiers were analysed using the WHO Health Inequality Toolkit. Inequalities were measured through difference (D), ratio (R), absolute concentration index (ACI), population attributable risk (PAR), and population attributable fraction (PAF), each with 95% confidence intervals (CIs).

**Results:**

The study found that the national ITN use increased from 3.9% in 2003 to 54.1% in 2019, before declining to 49.0% in 2022. Wealth-based inequality widened substantially, with the ACI shifting from − 0.3 (95% CI − 1.4 to 0.8) in 2003 to − 7.9 (95% CI − 8.8 to − 7.1) in 2022. The difference between the poorest and richest quintiles rose from − 4.1% in 2003 to − 39.4% in 2022, while the ratio declined from 0.8 to 0.4. Regional disparities were also marked, with the difference increasing from 21.8% in 2003 to 39.5% in 2022, and ratios rising from 2.2 to 2.3. Residence-based inequalities grew, with rural–urban difference widening from − 0.2% in 2003 to − 25.1% in 2022. Sex and age contributed minimally, with ACIs fluctuating (− 2.5 in 2014 and 1.0 in 2022) for sex.

**Conclusions:**

Although ITN use among Ghanaian children under five has increased substantially since 2003, widening inequities persist, particularly by wealth, region, and residence. Findings highlight the need for equity-focused policies to accelerate progress towards Sustainable Development Goal 3.3, ending malaria by 2030.

## Background

Malaria remains one of the leading causes of morbidity and mortality in children under 5 years, particularly in sub-Saharan Africa. Globally, malaria caused an estimated 249 million cases and 608,000 deaths in 2022, of which approximately 76% occurred in children under five [1, 2]. Insecticide-treated nets (ITNs) are recognised as one of the most effective malaria-prevention tools, reducing child mortality and morbidity substantially when coverage and consistent use are high [3]. Despite notable global gains, substantial inequities persist, with only 57.4–41.2% of children under five in endemic regions of sub-Saharan Africa reported to be sleeping under an ITN [4, 5].

In Ghana, trends mirror regional progress but show persistent disparities. ITN use among children under five increased from 58.1% in 2003 to 62.9% in 2022 [6], yet national averages obscure stark variations. Recent survey data reported that only 57.4% of under-five children in households with at least one ITN slept under a net, with utilisation markedly higher in rural (66.2%) compared to urban (43.5%) areas [5]. In addition, regional inequalities are evident, ranging from about 80.6% in Upper West to 30.5% in Greater Accra [5]. Such disparities underscore the importance of examining equity rather than focusing solely on overall prevalence.

Several sociodemographic factors influence ITN use among children under 5 years [4–8]. Evidence shows that sex differences are often modest [9], though in some contexts, male children are slightly more likely to be prioritised for net use [9]. Age plays a role, with younger children more likely to be placed under ITNs than older under-fives [4]. Place of residence consistently shapes access, with rural households often having higher ITN utilisation than urban households [3, 10], partly due to targeted mass distribution campaigns in rural zones. Regional variation reflects differing malaria transmission intensities, distribution effectiveness, and behavioural norms across Ghana’s 16 regions [11]. Economic status is a persistent determinant, with poorer households that may depend more heavily on free distribution programmes [8, 12, 13], whereas wealthier families sometimes underutilise ITNs, possibly because of alternative prevention measures or perceived lower vulnerability [14, 15].

The importance of addressing these inequities cannot be overstated. Children under five are highly vulnerable to severe malaria, anaemia, and long-term developmental consequences [16–18]. However, an unequal ITN use perpetuates preventable deaths, undermines Ghana’s progress in malaria control, and sustains broader health inequities [19]. In addition, understanding the role of sociodemographic determinants sex, age, residence, region, and wealth, in shaping disparities is essential for guiding interventions that reach the most vulnerable children and for ensuring equitable health outcomes [20, 21].

Despite Ghana’s concerted efforts in malaria control, including nationwide distribution campaigns of ITNs, there is insufficient evidence on how disparities in ITN use among children under five have evolved over two decades. Existing studies highlight utilisation of ITN [7, 22–24], but often fail to decompose inequalities into within- and between-group dimensions, limiting insight into which factors contribute most to persistent inequities. This knowledge gap constrains policy and practice, as interventions may not be adequately tailored to underserved groups. The purpose of this study is, therefore, to apply decomposition analysis, using the WHO Health Equity Toolkit, to assess disparities in ITN use among children under five in Ghana from 2003 to 2022. However, by systematically assessing inequalities across sex, age, place of residence, region, and economic status, this study aims to generate evidence that can guide the development of targeted and equity-oriented interventions. Findings will guide policy decisions on resource allocation, strengthen community-level health promotion strategies, and contribute to Ghana’s progress towards Sustainable Development Goal 3.3 to end malaria by 2030.

## Methods

### Study design and data sources

This study used secondary data collected through a cross-sectional design, drawing on six rounds of the nationally representative Ghana Demographic and Health Surveys (GDHS) conducted between 2003 and 2022. The data sets, accessed through the WHO HEAT, contain standardised and pre-cleaned information on household demographics, health behaviours, and malaria-prevention indicators, including ITN ownership and use. Each GDHS round employed a uniform two-stage stratified sampling design, ensuring methodological consistency across survey years and comparability of estimates over time. The study population comprised children under 5 years of age who were present in the household on the night preceding the survey to maintain accuracy in ITN-use reporting.

### Outcome variable and equity stratifiers

The primary outcome was ITN use among children aged 0–59 months, defined as whether a child was reported to have slept under an ITN on the night preceding the survey. The analysis was guided by equity stratifiers recommended by the WHO Health Inequality Data Repository and Toolkit, encompassing five key dimensions which include sex (male or female), child’s age (< 12 months, 12–23 months, 24–35 months, 36–47 months, and 48–59 months), place of residence (urban or rural), region (ten subnational regions before 2017 and sixteen in the 2022 survey), and economic status, which was categorised into household wealth quintiles derived from principal components analysis of household assets and amenities.

### Statistical analysis

Data cleaning and statistical analyses were performed using the WHO Health Inequality Toolkit (HEAT). Sampling weights provided in the DHS data sets were applied to ensure representativeness. Descriptive analyses were conducted to summarise trends in ITN use across survey years and stratifiers. Inequality was quantified using both simple and complex measures. Simple measures included the difference (D) and ratio (R) between the most-advantaged and least-advantaged subgroups. Complex measures comprised the population attributable fraction (PAF), population attributable risk (PAR), and Absolute Concentration Index (ACI), which take into account all subgroup data and population distribution. Ninety-five percent confidence intervals (95% CI) were calculated for each measure.

### Inequality measures and sign conventions

We computed absolute and relative measures recommended by WHO HEAT: difference (D), ratio (R), absolute concentration index (ACI), population attributable risk (PAR), and population attributable fraction (PAF). For ordered dimensions (wealth quintile), ACI summarises the distribution across groups, negative ACI indicates a pro-poor distribution (higher use among the worse-off), and positive ACI indicates a pro-rich distribution. PAR and PAF are computed relative to the best-performing subgroup (counterfactual). All measures are scaled × 100 for interpretability.

### Survey design and estimation

National ITN use rose substantially from 2003 to 2019, followed by a modest decline in 2022. Inequalities widened for wealth and residence, with higher use among poorer and rural households (pro-poor) across most years. Regional gaps persisted. All estimates used DHS/MIS sampling weights and accounted for clustering and stratification (primary sampling units and strata) in variance estimation. We derived 95% confidence intervals using Taylor linearization and report uncertainty for D, R, ACI, PAR, and PAF where available.

### Regional harmonisation and comparability

Ghana transitioned from 10 to 16 administrative regions between survey rounds. To ensure comparability, we harmonised regions to a consistent scheme for trend analyses or reported pre/post separately, where harmonisation was infeasible. We also considered the survey month/season to contextualise year-to-year variation.

### Secondary indicator

As a behavioural indicator, we propose reporting ‘use among those with access’ (UAA) alongside overall use, to separate availability from use, this can clarify urban/wealth gradients.

### Ethical considerations

Analyses used de-identified microdata from the Ghana Demographic and Health Surveys. The DHS Program obtains informed consent from all participants, and protocols are approved by the Ghana Statistical Service Ethics Committee and the ICF Institutional Review Board. This secondary analysis of public-use data was exempt from additional review.

## Results

### Trends and inequalities in ITN use among children under 5 years in Ghana by national, 2003–2022

**Fig. 1:**
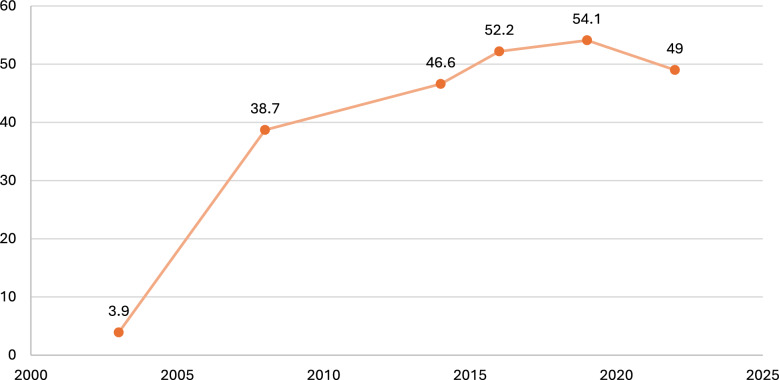
Trends and inequalities in ITN use among children under 5 years in Ghana by national, 2003–2022

The national trend of ITN use among children under 5 years in Ghana shows a remarkable increase between 2003 and 2019, followed by a slight decline in 2022. In 2003, coverage was extremely low at just 3.9%. Substantial progress was achieved by 2008 (38.7%), reflecting the impact of early large-scale distribution campaigns. Uptake continued to rise steadily, reaching 46.6% in 2014 and surpassing 50% by 2016 (52.2%) and 2019 (54.1%). However, by 2022, coverage declined modestly to 49%, suggesting challenges in maintaining earlier gains (Fig. 1).

### Trends and regional inequalities in ITN use among children under 5 years in Ghana by sex and subnational region, 2003–2022

**Fig. 2:**
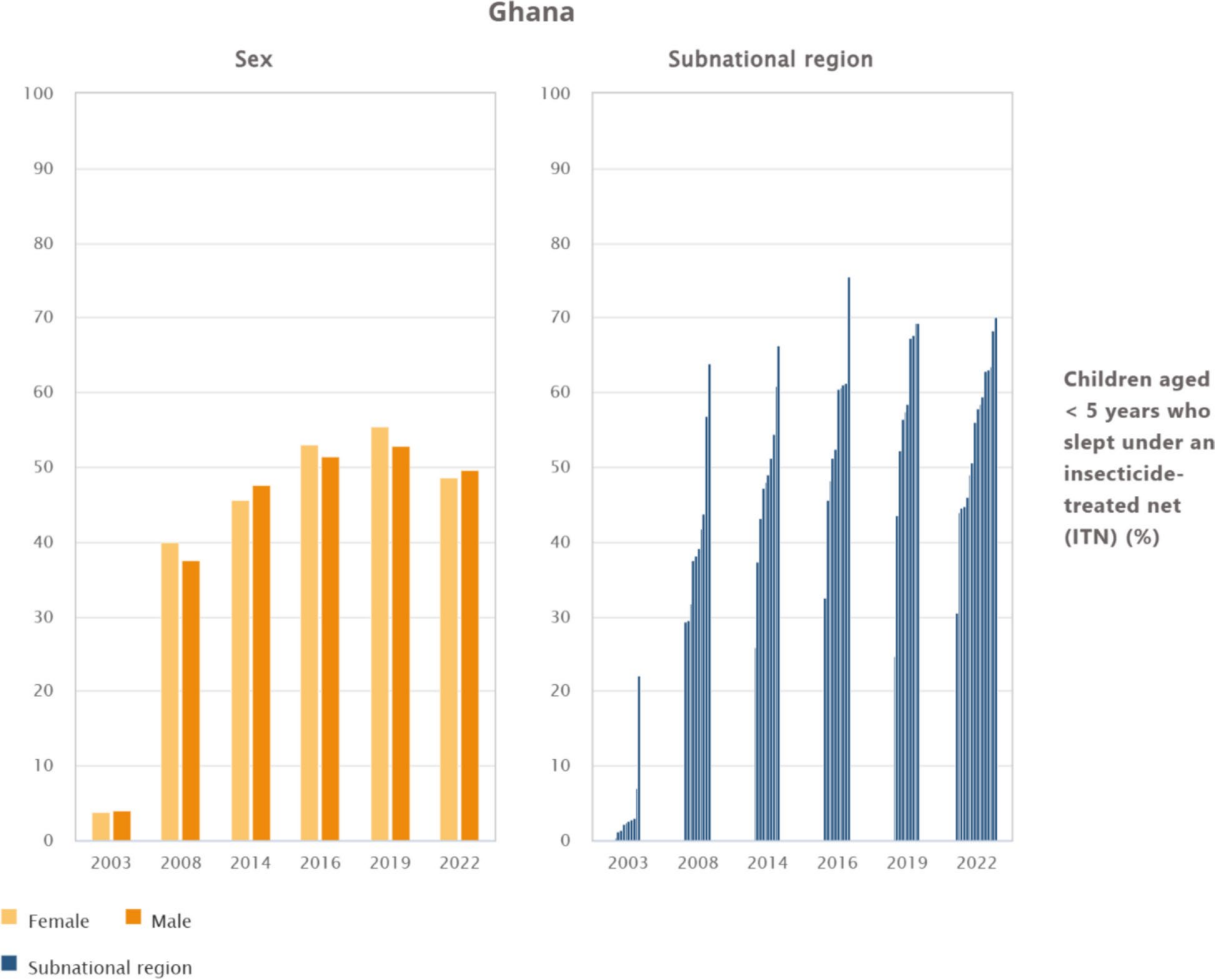
Trends and inequalities in ITN use among children under 5 years in Ghana by sex and subnational region, 2003–2022

Figure 2 presents trends in ITN use among children under 5 years in Ghana, showing minimal gender disparities but persistent regional inequalities. In terms of sex, the use of ITNs among children under 5 years in Ghana shows minimal variation between males and females. From negligible levels in 2003 (3.8% for females and 4% for males), coverage rose to around 40% by 2008 and exceeded 50% in 2014–2019. Despite a slight decline in 2022 (48.5% for females and 49.5% for males), near parity was maintained. By contrast, regional differences are substantial. In 2003, the Upper East region recorded relatively high use (22.1%), while most other regions reported rates below 5%. By 2008, the Upper West (63.8%) and Brong Ahafo (56.9%) had emerged as leaders, whereas Greater Accra and Central remained below 30%. The inequalities persisted through 2014–2019, with Upper East (75.5%) and Upper West (69.3%) maintaining the highest uptake, while Greater Accra lagged far behind (24.7–32.6%). The 2022 data, reflecting new administrative boundaries, further underscore these disparities with Oti (70.1%), Ahafo (68.3%), and Savannah (63.5%) reported the highest coverage, while Greater Accra remained the lowest at 30.6%.

### Trends and inequalities in ITN use among children under 5 years in Ghana by age, wealth quintile, and place of residence, 2003–2022

**Fig. 3:**
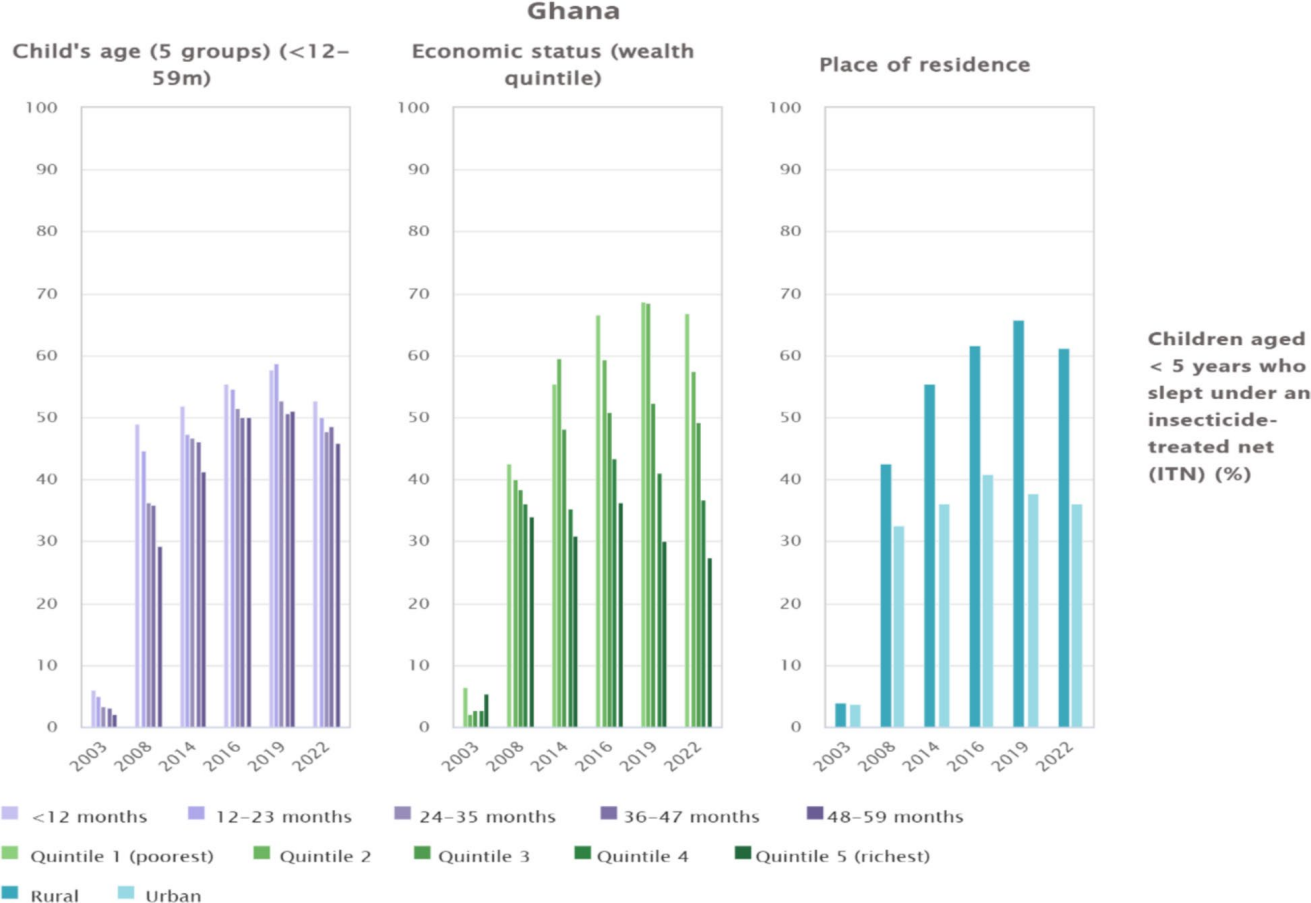
Trends and inequalities in ITN use among children under 5 years in Ghana by age, wealth quintile, and place of residence, 2003–2022

Figure 3 illustrates the evolution of ITN use among children under 5 years in Ghana, disaggregated by age, economic status, and residence. ITN use increased substantially from below 10% in 2003 to above 50% in most subgroups by 2014–2019, although a decline was observed in 2022. Younger children, particularly those under 12 months, consistently exhibited higher coverage compared with older age groups, though all groups recorded lower usage in the most recent survey. Clear wealth-related inequalities were also observed, with children from the poorest quintile reporting consistently higher ITN use than those from the richest quintile, and the disparity widening markedly in 2022 (66.8% vs. 27.4%). Rural–urban gaps persisted throughout the period, with rural children showing greater ITN coverage (61.1%) compared to their urban counterparts (36%) in 2022.

### Inequality of factors associated with insecticide-treated net use among children under 5 years in Ghana by different inequality dimensions, 2003–2022

For economic status, the absolute concentration index (ACI) shifted from − 0.3 (95% CI − 1.4 to 0.8) in 2003 to − 7.9 (95% CI − 8.8 to − 7.1) in 2022, indicating a consistent pro-poor distribution. The difference (D) widened substantially from − 4.1 in 2003 to − 39.4 in 2022, while the ratio (R) declined from 0.8 to 0.4, underscoring intensifying inequities between the poorest and richest quintiles. For the subnational region, disparities were even greater, with the difference rising from 21.8 in 2003 to 39.5 in 2022, and the ratio increasing from 2.2 to 2.3, pointing to marked geographic variation in coverage. Residence-based inequalities also persisted, as the difference widened from − 0.2 in 2003 to − 25.1 in 2022, while the ratio declined from 1.0 to 0.6. In contrast, children’s age contributed little to inequality, the ACI ranged from − 0.8 (95% CI − 1.0 to − 0.6) in 2003 to − 1.7 (95% CI − 2.6 to − 0.7) in 2022, with negligible attributable risks. Similarly, sex showed no systematic pattern, with the ACI fluctuating around zero (− 2.5 in 2014; 1.0 in 2022), and differences remaining small across survey years (see Table 1).

**Table 1:** Inequality of factors associated with insecticide-treated net (ITN) use among children under 5 years in Ghana by different inequality dimensions, 2003–2022

		2003			2008			2014			2016			2019			2022		
Dimension	Measure	Est. (%)	CI–LB	CI–UB	Est. (%)	CI–LB	CI–UB	Est. (%)	CI–LB	CI–UB	Est. (%)	CI–LB	CI–UB	Est. (%)	CI–LB	CI–UB	Est. (%)	CI–LB	CI–UB
Economic status (wealth quintile)	ACI	− 0.3	− 1.4	0.8	− 1.7	− 2.6	− 0.7	− 5.7	− 8.5	− 2.9	− 6.1	− 6.3	− 5.9	− 8.0	− 10.6	− 5.4	− 7.9	− 8.8	− 7.1
	D	− 4.1	NA	NA	− 10.7	NA	NA	− 24.4	NA	NA	− 30.3	NA	NA	− 38.5	NA	NA	− 39.4	NA	NA
	PAF	37.4	36.9	37.8	64.8	64.6	64.9	42.2	42.1	42.3	44.6	44.5	44.8	28.0	27.9	28.1	43.2	43.1	43.3
	PAR	1.5	− 0.2	3.2	25.1	17.8	32.4	19.7	15.5	23.9	23.3	15.5	31.0	21.1	15.8	26.5	21.1	15.8	26.5
	R	0.8	NA	NA	0.6	NA	NA	0.6	NA	NA	0.5	NA	NA	0.4	NA	NA	0.4	NA	NA
Child’s age (5 groups) (< 12–59 m)	ACI	− 0.8	− 1.0	− 0.6	− 3.9	− 4.8	− 3.0	− 1.2	− 1.7	− 0.7	− 1.7	− 2.6	− 0.7	− 1.7	− 2.6	− 1.1	− 1.2	− 1.7	− 0.7
	D	− 4.1	NA	NA	− 19.8	NA	NA	− 10.7	NA	NA	− 5.4	NA	NA	− 6.6	NA	NA	− 6.7	NA	NA
	PAF	0.0	− 0.3	0.3	0.0	− 0.1	0.1	0.0	− 0.1	0.1	0.0	− 0.1	0.1	0.0	− 0.1	0.0	0.0	− 0.0	0.0
	PAR	0.0	− 0.9	1.0	0.0	− 3.6	3.6	0.0	− 3.6	3.6	0.0	− 3.4	3.4	0.0	− 2.5	2.5	0.0	− 2.1	2.1
	R	0.3	NA	NA	0.6	NA	NA	0.9	NA	NA	0.9	NA	NA	0.9	NA	NA	0.9	NA	NA
Place of residence	D	− 0.2	NA	NA	− 10.0	NA	NA	− 19.3	NA	NA	− 20.9	NA	NA	− 27.9	NA	NA	− 25.1	NA	NA
	PAF	0.0	− 0.2	0.2	0.0	− 0.0	0.0	0.0	− 0.0	0.0	0.0	− 0.0	0.0	0.0	− 0.0	0.0	0.0	− 0.0	0.0
	PAR	0.0	− 0.9	0.9	0.0	− 1.6	1.6	0.0	− 1.4	1.4	0.0	− 1.9	1.9	0.0	− 2.1	2.1	0.0	− 1.1	1.1
	R	1.0	NA	NA	0.8	NA	NA	0.8	NA	NA	0.7	NA	NA	0.6	NA	NA	0.6	NA	NA
Sex	D	0.2	NA	NA	− 2.5	NA	NA	1.9	NA	NA	− 1.6	NA	NA	− 2.5	NA	NA	1.0	NA	NA
	PAF	2.5	2.4	2.7	− 3.2	− 3.1	− 3.0	1.9	1.9	1.9	− 0.0	− 0.0	− 0.0	− 0.0	− 0.0	0.0	1.0	1.0	1.0
	PAR	0.1	− 0.5	0.7	− 1.2	− 1.2	− 0.2	0.9	− 0.3	2.1	0.9	− 1.7	1.7	0.9	− 1.8	2.1	0.5	− 0.6	2.1
	R	1.1	NA	NA	1.0	NA	NA	1.0	NA	NA	1.0	NA	NA	1.0	NA	NA	1.0	NA	NA
Subnational region	D	21.8	NA	NA	34.4	NA	NA	40.4	NA	NA	42.9	NA	NA	44.6	NA	NA	39.5	NA	NA
	PAF	465.3	464.1	466.4	64.8	64.6	64.9	42.2	42.1	42.3	44.6	44.5	44.8	28.0	27.9	28.1	43.2	43.1	43.3
	PAR	18.2	13.6	22.7	25.1	17.8	32.4	19.7	15.5	23.9	23.3	15.5	31.0	21.1	15.8	26.5	21.1	15.8	26.5
	R	73.7	NA	NA	2.2	NA	NA	2.2	NA	NA	2.3	NA	NA	2.8	NA	NA	2.3	NA	NA

All estimates are weighted; 95% CIs reflect complex survey design

D, absolute difference between most- and least-advantaged subgroups; R, ratio; ACI, absolute concentration index (× 100; negative = pro-poor); PAR, population attributable risk (percentage points); PAF, population attributable fraction (%), bounded between − 100% and + 100%; reference = best-performing subgroup

## Discussion

### Summary of findings

The findings reveal substantial progress in ITN use among children under 5 years in Ghana, with national coverage rising from 3.9% in 2003 to 54.1% in 2019, before declining slightly to 49% in 2022. While gender differences remained negligible throughout the period, significant regional, socioeconomic, and residence-based inequalities persisted. Northern regions such as Upper East and Upper West consistently recorded the highest coverage, contrasting with Greater Accra, which remained the lowest, particularly in 2022 (30.6%). Younger children, rural residents, and those from the poorest households were more likely to use ITNs, with the poorest quintile (66.8%) far exceeding the richest (27.4%) in 2022. Inequality measures confirm these disparities, with economic status and residence showing widening pro-poor inequities, whereas age and sex contributed minimally. These findings affirm the importance of equity-focused interventions to sustain gains and close persistent gaps in ITN use.

This study used nationally representative data to examine the trends and inequalities in ITN use among children under 5 years in Ghana from 2003 to 2022. We found that ITN use increased substantially from 3.9% in 2003 to 54.1% in 2019, before declining modestly to 49% in 2022. Across the two decades, sex- and age-related inequalities were negligible, whereas substantial disparities persisted and widened by economic status, residence, and region. Ghana achieved remarkable progress between 2003 and 2019, reflecting the impact of large-scale mass distribution campaigns and health promotion efforts. However, the subsequent decline in 2022 highlights challenges in sustaining coverage. Similar patterns of stalled or declining utilisation after earlier gains have been reported across sub-Saharan Africa, with the World Malaria Report 2022 attributing these setbacks to supply chain disruptions, funding shortfalls, and behavioural fatigue [25]. In Ghana, the Malaria Behaviour Survey 2022 further noted that although household ownership remained high, the use-to-access ratio was only 0.65, underscoring persistent behavioural barriers [26].

Economic inequalities were striking. The absolute concentration index (ACI) shifted from − 0.3 in 2003 to − 7.9 in 2022, while the difference (D) widened from − 4.1 to − 39.4, confirming a strong pro-poor distribution. By 2022, 66.8% of children in the poorest quintile slept under ITNs compared with only 27.4% in the richest quintile. This is consistent with findings from Ghana and Nigeria, which show that poorer households often exhibit higher ITN use, partly due to residence in high-transmission zones and reliance on free distribution programmes [7, 27]. The pro-poor pattern of ITN use observed aligns with Ghana’s National Malaria Elimination Programme strategy, which prioritises free distribution in high-transmission and economically disadvantaged areas. This suggests that the programme is effectively reaching those most at risk. Nonetheless, lower utilisation among wealthier and urban households may also reflect differences in perceived need, reliance on alternative prevention methods, and reduced malaria exposure due to better housing and environmental conditions [23]. While these contextual factors partly justify the observed pattern, achieving universal protective coverage requires addressing behavioural barriers and maintaining consistent use across all groups to prevent residual transmission.

Residence-based inequalities also persisted. In 2022, 61.1% of rural children used ITNs compared with only 36% in urban areas, with the difference (D) rising to − 25.1 and the ratio (R) falling to 0.6. This urban disadvantage mirrors findings from Ghana and Mozambique, where urban households often cite barriers, such as heat, crowding, and lower perceived malaria risk [28, 29]. Tailored social and behaviour change interventions, coupled with innovations in net design, are, therefore, required to increase urban uptake. Regional disparities were equally pronounced. Coverage in the northern ecological zones (Upper East, Upper West, and later Oti and Savannah) consistently exceeded national averages, while Greater Accra remained the lowest throughout the period. The regional difference widened from 21.8 percentage points in 2003 to 39.5 in 2022, with the ratio (R) rising from 2.2 to 2.3. Similar subnational inequalities have been observed across Africa, often reflecting differential programme targeting and ecological malaria risk [30]. This suggests that sustained investment in urban and coastal regions is essential to achieving equity.

By contrast, age- and sex-based inequalities were minimal. The ACI for age was − 0.8 in 2003 and − 1.7 in 2022, while sex fluctuated around zero. Although infants under 12 months were slightly more likely to use ITNs, all age groups experienced declines by 2022, indicating that systemic factors rather than intra-household preferences drive the overall trend. The absence of systematic sex differences aligns with prior studies showing that once ITNs are available, allocation among boys and girls is equitable [31]. While Ghana achieved major gains in ITN coverage between 2003 and 2019, widening socioeconomic, residence, and regional inequalities in 2022 highlight persistent barriers to universal coverage. To sustain progress, equity-sensitive policies are needed, with particular emphasis on improving uptake among urban and wealthier households. Strengthening continuous distribution, investing in durable nets, and implementing locally tailored behaviour change programmes will be critical to achieving the malaria control and child survival targets of Sustainable Development Goal 3.2.

### Policy, practice, and research implications

The findings reveal that despite remarkable improvements in ITN coverage among children under five in Ghana, inequities remain entrenched, particularly across economic status, residence, and region. Stakeholders such as the Ministry of Health (MoH), Ghana Health Service (GHS), and the National Malaria Elimination Programme (NMEP) must work collaboratively to sustain coverage gains and close persistent gaps. National policymakers should strengthen equity-sensitive distribution strategies, ensuring consistent supply chains and targeted campaigns in underperforming regions such as Greater Accra. Partnerships with district health directorates, metropolitan and municipal assemblies, and international partners, including the Global Fund, WHO, and UNICEF, will be essential in providing financial, logistical, and technical support. Policymakers should also integrate malaria prevention into broader child health and social protection programmes, ensuring that equity is central to national strategies for achieving SDG 3.2.

Frontline implementation rests heavily on regional and district health authorities, community health officers, and community-based volunteers who directly engage with households. Sustained behaviour change communication strategies should be reinforced through antenatal care clinics, child welfare clinics, and schools, promoting ITN use beyond infancy and addressing the declining uptake observed in older children. Collaboration with traditional leaders, faith-based organisations, and civil society groups will enhance culturally sensitive health messaging and address perceptions that reduce uptake in urban and wealthier households. In addition, the private sector, especially pharmaceutical outlets and retail distributors, should be engaged in ITN promotion and distribution, ensuring accessibility beyond periodic national campaigns.

Academic institutions, including schools of public health and nursing faculties, together with national research bodies, play a pivotal role in generating evidence on the behavioural, socio-cultural, and system-level determinants of ITN utilisation. Strengthening collaborations with international research partners, universities, and donor-supported consortia will enhance methodological rigour and promote comparative analyses across different contexts. Future research should prioritise identifying barriers to sustained ITN use among urban, wealthier, and older–child subgroups, assessing the effectiveness of net replacement and continuous distribution mechanisms, and evaluating integrated malaria-prevention approaches. Research outputs should be translated into concise policy briefs and disseminated through partnerships with the MoH, GHS, and development partners to ensure that evidence directly informs practice and long-term malaria elimination strategies.

### Strengths and limitations

This study draws strength from the use of nationally representative survey data spanning nearly two decades, enabling a robust assessment of long-term trends in ITN use among children under five in Ghana, with consistent methodologies ensuring comparability across survey years. The application of multiple inequality measures from the WHO Health Inequality Toolkit provides a comprehensive understanding of disparities across wealth, region, residence, age, and sex, generating policy-relevant evidence. However, limitations include reliance on self-reported ITN use, which may be subject to recall or social desirability bias, and the cross-sectional nature of the data, which restricts causal inference. The reconfiguration of administrative boundaries in 2022 also constrains comparability with earlier subnational analyses, while unmeasured factors such as maternal education and household decision-making were not fully captured. In addition, findings should be interpreted considering potential seasonal differences in fieldwork timing across survey rounds, changes in regional administrative boundaries, and the reliance on ‘slept under an ITN last night’ without simultaneously reporting access. Self-report may also introduce desirability bias. These factors may affect the comparability of point estimates across years and subgroups. Furthermore, the analysis focused on reported use rather than ownership vs. consistent nightly utilisation, potentially masking critical gaps in effective ITN coverage.

## Conclusion

The use of ITNs among children under 5 years in Ghana rose markedly from 3.9% in 2003 to 54.1% in 2019, demonstrating the impact of nationwide distribution campaigns and malaria control efforts. A slight decline to 49% in 2022, however, signals the need for renewed commitment to sustaining coverage. Although gender disparities remain minimal, inequities persist across wealth, residence, and regional lines, with rural and poorer households maintaining higher use than their urban and wealthier counterparts. To address these gaps, government and policymakers should strengthen continuous distribution and replacement systems, design equity-driven interventions to narrow regional and socioeconomic divides, and promote culturally tailored health education initiatives. Enhanced monitoring and evaluation frameworks are also essential to guide resource allocation and assess progress. By implementing these measures and advancing research on equity in malaria prevention, Ghana can safeguard earlier gains, ensure fair access to ITNs, and reduce the burden of malaria among children under five.

## Data Availability

The data set used can be accessed at https://www.who.int/data/inequality-monitor/assessment_toolkit.

## Abbreviations

ACI: Absolute Concentration Index
CI: Confidence Interval
D: Difference
DHS: Demographic and Health Survey
GHS: Ghana Health Service
HEAT: Health Inequality Assessment Toolkit
ICF: Inner City Fund (Institutional Review Board)
ITN: Insecticide-Treated Net
MoH: Ministry of Health
NMEP: National Malaria Elimination Programme
PAF: Population Attributable Fraction
PAR: Population Attributable Risk
R: Ratio
SDG: Sustainable Development Goal
UNICEF: United Nations International Children’s Emergency Fund
WHO: World Health Organisation
